# Noninvasive Brain Stimulation for Neurorehabilitation in Post-Stroke Patients

**DOI:** 10.3390/brainsci13030451

**Published:** 2023-03-06

**Authors:** Kun-Peng Li, Jia-Jia Wu, Zong-Lei Zhou, Dong-Sheng Xu, Mou-Xiong Zheng, Xu-Yun Hua, Jian-Guang Xu

**Affiliations:** 1 School of Rehabilitation Science, Shanghai University of Traditional Chinese Medicine, Shanghai 201203, China; 2 Center of Rehabilitation Medicine, Yueyang Hospital of Integrated Traditional Chinese and Western Medicine, Shanghai University of Traditional Chinese Medicine, Shanghai 200437, China; 3 Department of Epidemiology, School of Public Health, Fudan University, Shanghai 200032, China; 4 Department of Traumatology and Orthopedics, Yueyang Hospital of Integrated Traditional Chinese and Western Medicine, Shanghai University of Traditional Chinese Medicine, Shanghai 200437, China; 5 Engineering Research Center of Traditional Chinese Medicine Intelligent Rehabilitation, Ministry of Education, Shanghai 201203, China

**Keywords:** noninvasive brain stimulation, repetitive transcranial magnetic stimulation, transcranial direct current stimulation, transcranial focused ultrasound stimulation, transcutaneous vagus nerve stimulation, post-stroke

## Abstract

Characterized by high morbidity, mortality, and disability, stroke usually causes symptoms of cerebral hypoxia due to a sudden blockage or rupture of brain vessels, and it seriously threatens human life and health. Rehabilitation is the essential treatment for post-stroke patients suffering from functional impairments, through which hemiparesis, aphasia, dysphagia, unilateral neglect, depression, and cognitive dysfunction can be restored to various degrees. Noninvasive brain stimulation (NIBS) is a popular neuromodulatory technology of rehabilitation focusing on the local cerebral cortex, which can improve clinical functions by regulating the excitability of corresponding neurons. Increasing evidence has been obtained from the clinical application of NIBS, especially repetitive transcranial magnetic stimulation (rTMS) and transcranial direct current stimulation (tDCS). However, without a standardized protocol, existing studies on NIBS show a wide variation in terms of stimulation site, frequency, intensity, dosage, and other parameters. Its application for neurorehabilitation in post-stroke patients is still limited. With advances in neuronavigation technologies, functional near-infrared spectroscopy, and functional MRI, specific brain regions can be precisely located for stimulation. On the basis of our further understanding on neural circuits, neuromodulation in post-stroke rehabilitation has also evolved from single-target stimulation to co-stimulation of two or more targets, even circuits and the network. The present study aims to review the findings of current research, discuss future directions of NIBS application, and finally promote the use of NIBS in post-stroke rehabilitation.

## 1. Introduction

Stroke is the second-leading cause of death and the third leading cause of disability in the world, and patients with stroke often suffer from functional impairments and deficits [1]. Motor weakness, sensory dysfunction, speech disturbances, dysphagia, unilateral neglect, cognitive dysfunction, and emotional impairment in post-stroke patients will need long-term rehabilitation [2,3,4]. The pathology of stroke involves both the focal neurologic deficits and the impairment of the neural circuit or brain networks [5,6].

Physical therapy includes two types of treatments, of which one is physiotherapy, such as functional exercise and movement therapy [7]. The other one is physical factor therapy, a rehabilitation treatment employing physical stimulation such as heat, electricity, and magnetism [8,9,10]. Physical therapy is commonly used in stroke rehabilitation and has been demonstrated to improve brain plasticity after focal neurological impairment [11]. In addition, previous evidence has demonstrated positive effects of physical therapy on treating depression [12], schizophrenia [13], and Parkinson’s disease [14]. As a means of physical factor therapy, noninvasive brain stimulation (NIBS) is a physical therapy that can regulate specific regions in brain by electrical, magnetic, or ultrasound stimulation in vivo, thereby modulating the excitability of neurons and multiple brain functions [13,15,16,17]. 

The effects of NIBS on physical functions in post-stroke patients have been reported by extensive clinical research [18,19]. However, due to the small sample size (less than 30 individuals) and unstandardized stimulation parameters of clinical studies [20,21,22], NIBS has not yet been included in the standardized treatment protocols for post-stroke rehabilitation. Several systematic reviews, meta-analyses, and even guidelines have summarized the application of NIBS. However, little attention has been paid to the evolution of its modalities [23,24], namely, paradigms or protocols [20,21,25,26,27,28,29,30,31]. 

A review providing a valuable overview of different stimulation parameters of NIBS, such as stimulation site, intensity, duration, frequency, and mode, is necessary and meaningful to promote its application in post-stroke rehabilitation. These were lacking in a previous article by Shen et al. [32]. In the present study, we aim to summarize the advantages of different stimulation modalities, the basic principles of the four NIBS techniques, and their current application in post-stroke dysfunction. Furthermore, the potential trend of NIBS-related research is further presented.

## 2. Search Strategy and Selection Criteria

The published studies cited in this review were published between 2000 and 2022, with a major focus on the years 2017 to 2022. The PubMed database was used to search relevant studies with the following keywords: non-invasive brain stimulation, repetitive transcranial magnetic stimulation, transcranial direct current stimulation, transcranial focused ultrasound stimulation, transcutaneous vagus nerve stimulation, post-stroke, neurorehabilitation, and dysfunction. All references were cited to the content-related parts of the review.

## 3. Overview of Neuromodulation and NIBS

Neuromodulation technology refers to the use of implantable or non-implantable techniques (e.g., electrical, magnetic, or ultrasonic methods) to obtain therapeutic effects by changing the function or state of the nervous system. Through this approach, neurons or nerve signal transduction in adjacent or distant parts of the stimulation site are excited, inhibited, or regulated, thereby changing nerve function and improving the quality of life of patients [15,18,19]. Noninvasive neuromodulation techniques mainly include transcranial magnetic stimulation (TMS), transcranial electrical stimulation (tES), transcranial focused ultrasound stimulation (tFUS), transcranial unfocused ultrasound stimulation (tUUS), and transcutaneous vagus nerve stimulation (tVNS), among which repetitive transcranial magnetic stimulation (rTMS) and tDCS have proven effective in treating depression and pain [33,34,35]. Due to the specific advantages of being noninvasive, painless, safe, and cheap, in addition to having different parameters and treatment modes, NIBS shows broad prospects for development.

### 3.1. Technology and Research Situation of NIBS

#### 3.1.1. TMS

TMS induces electrical currents in the brain through electromagnetic induction caused by an energized coil placed on the scalp that is a highly effective, painless, and noninvasive brain stimulation procedure, causing changes in excitability and plasticity of the targeted cortical neuronal populations. Magnetic fields can penetrate the scalp and skull and generate subthreshold- or suprathreshold-induced currents in the cerebral cortex concurrently, which depolarize neurons to generate action potentials to modulate and stimulate neuronal activity in target areas, and then can mostly affect the cortex function by synchronizing activity in related brain regions [22]. The regulatory effect of TMS on the cerebral cortex is influenced by factors such as coil shape, stimulation site, frequency, intensity, dosage (number of pulses, time, and duration) and other parameters. 

A target for TMS stimulation refers to the location where the magnetic field acts, also known as “stimulation target area or action (treatment) target area”. The primary motor cortex (M1) and dorsolateral prefrontal cortex (DLPFC) are more common targets for stimulation in clinical practice and research [36]. The dorsal premotor region (dPM), subthalamic nucleus (STN), dorsal anterior cingulate cortex (dACC), primary somatosensory cortex (S1), and supplementary and pre-supplementary motor areas complex (SMA proper and pre-SMA) are also frequently used as targets [37]. According to the navigation of functional magnetic resonance imaging (fMRI) results, the precise stimulation of brain regions with abnormal functional connectivity in stroke patients by rTMS can be achieved, and more targets that match individual anatomy or functional physiology can be presented [38,39].

TMS includes single-pulse (sTMS), paired-pulse TMS (pTMS), and rTMS according to the stimulation mode. sTMS means that only one stimulation pulse is issued per stimulation cycle without long-term regulatory effects, and is often used in the detection of neural pathways [40]. pTMS denotes that two stimuli separated by variable intervals can be sent out in each stimulation cycle, which is commonly used in the study of cortical excitability [41]. rTMS refers to the continuous pulse of a specific frequency sent out in each stimulation cycle, which is divided into low frequency (LF-rTMS, ≤1 Hz) and high frequency (HF-rTMS, >1 Hz). LF- and HF-rTMS show different regulatory effects on the cerebral cortex. Generally, HF-rTMS (especially ≥5 Hz) can increase cortical excitability, while LF-rTMS decreases it [42]. Theta burst stimulation (TBS) is another modality delivering 3–8 Hz rhythmic pulse of HF-rTMS, in which continuous theta burst stimulation (cTBS) shows inhibitory effects and intermittent theta burst stimulation (iTBS) shows excitatory effects [43]. Paired associative stimulation (PAS) is a stimulation program that combines rTMS and electrical stimulation [37], which acts primarily on the central nervous system and on the peripheral nervous system, respectively. They were conducted at specific time intervals to enhance the effects of rTMS and induce lasting changes in the excitability of the motor system [44].

Another factor that affects the effect of rTMS is the variation of stimulation parameters, such as frequency (ranging from 5 Hz to 20 Hz), intensity (ranging from 80% to 115% of resting motor threshold, RMT), and total number of pulses (ranging from 120 to 2500). A systematic review concluded that higher stimulation frequency (>5 Hz), more stimulation (>500), and multiple sessions (>1) yielded better results in improving cognitive function for individuals with neurological diseases, such as attention, executive functions, and working memory [45]. It has also been reported that multistage rTMS can produce long-term effects lasting up to 6 months [46]. Other influencing factors [47], including coil orientation, duration of each pulse sequence, inter-stimulus interval, number of pulse sequences, and duration of follow-up, still need further investigation.

Since the first device reported by Barker et al. in 1985, rTMS has been widely used in the treatment of pain, Parkinson’s disease, stroke, cognitive dysfunction, multiple sclerosis, depression, anxiety, and other disorders (schizophrenia, substance addiction, and craving) [22,48]. A prior study confirmed the safety of rTMS in the treatment of neurological disorders [49]. By applying different modes and parameters of stimulation to different targets, rTMS can regulate the excitability of local neuron clusters, thereby modulating the excitability of the neural circuits and networks in which they are located, ultimately improving brain function.

#### 3.1.2. tES

tES refers to physical therapy using electrical current to stimulate the brain, and it has gained popularity as a long-term therapy for patients with neurological disorders due to its convenience and potential effects on the brain network [50]. tES is an NIBS technique in experimental and clinical fields, including transcranial direct current stimulation (tDCS), transcranial alternating current stimulation (tACS), transcranial pulsed current stimulation (tPCS), and transcranial random noise stimulation (tRNS). Unlike tACS and tDCS, tPCS can intrigue randomly generated quadratic pulses, which may in turn increase endogenously generated brain oscillations, thereby facilitating the synchronization of deep brain structures with cortical activity [51]. Another promising alternative, tRNS, is a noisy electrical stimulation that increases cortical excitability through a random “noise” resonance [52]. Both of them are currently lacking in research, herein, the two most widely used tES techniques, tDCS and tACS, are summarized in the following section.

##### tDCS 

tDCS was reported for use in the intact human as an NIBS technique in 2000 [53]. It mainly delivers low direct current to brain tissue through electrodes placed on the scalp. A constant electric field can impact the neurons in the cerebral cortex, thereby activating sodium–potassium pumps, calcium-dependent channels, and NMDA receptor activity to depolarize or hyperpolarize neuronal membrane potential [54,55], thus regulating neuronal activity and cortical excitability. However, unlike TMS, tDCS does not induce action potentials directly, whereby stimulation sites are more scattered. Its effects are thought to be achieved by altering the polarity of the membrane to change their probability of triggering action potentials, thus modulating the activation of neurons in the stimulated area. The parameters of tDCS have a decisive effect on its biological effects [56], including electrode shape, size, number, placement, polarity, stimulation intensity, duration, and stimulation waveform [57,58], in which slight changes will produce distinctly different effects [59]. The spatial resolution and stimulation depth of tDCS are at the centimeter level (1 cm); therefore, the cerebral cortex with specific functions is often selected as the stimulation target area. For most clinical studies, the placement of the electrode is generally determined by the international convention of the EEG 10/20 system. The area of the electrodes used ranges from 25 to 35 cm^2^. In most cases, anodal tDCS depolarizes the resting membrane potential of neurons, thereby increasing the rate of spontaneous neuronal discharge and increasing cortical excitability. Common anodal electrode locations include the primary motor cortex, left dorsolateral prefrontal, and occipital lobes. In contrast, cathodal tDCS decreases neuronal excitability by shifting the resting membrane potential toward hyperpolarization. Cathodal electrode locations including the right supraorbital area, the central region, and the arm were commonly used [60]. Unihemispheric and bihemispheric montages as a way to optimize electrode placement ensure that specific brain regions receive the desired dose of current [61]. Yu et al. [62] suggested cathodal ctDCS stimulation can improve the language and motor dysfunction for post-stroke patients. Notably, the effects may vary across stimulation sites. The current intensity of tDCS is usually 0.5–4 mA (most commonly 1–2 mA). There is no strict restriction on the duration of tDCS stimulation. A duration of 5–30 min is used in most cases, with 20 min being the most effective, while the interval between sessions is at least 48 h [59]. The waveform of tDCS, reported by Hsu G et al. [57], is divided into square wave, sharp wave, and sinusoidal wave, depending on whether the current is constant or pulsed [58]. tDCS also has a post-stimulus effect, with changes in cortical excitability lasting up to 1 h after stimulation if the stimulus duration is long enough. In addition to the immediate effect on membrane potential polarity, tDCS can modulate the synaptic microenvironment, such as altering NMDA receptor or GABA activity. It thus acts as a post-effect of modulating synaptic plasticity within the cortex (similar to long-term facilitation). Studies have found that a sustained increase in postsynaptic excitatory potentials can be observed with anodal stimulation of the motor cortex [63,64]. In addition, tDCS modulates excitability changes in the connected distal septal cortex and subcortical areas. Anodal stimulation of the left-hemisphere M1 premotor cortex area not only affects the corticospinal circuits involving the generation of motor-evoked potentials but also modulates transcallosal inhibition in the contralateral hemisphere via inhibitory interneurons.

##### tACS

tACS is a noninvasive form of tES using low-intensity alternating current. It selectively enhances synaptic connectivity in neuronal circuits and synaptic plasticity by modulating the brain’s intrinsic endogenous neural oscillatory patterns and altering neurotransmitter levels [65]. Then, tACS ultimately improves the coordinated activity between local brain areas and brain regions [50,66]. Unlike TMS and tDCS, tACS completely avoids skin irritations [50]. It stimulates cortical neurons with sinusoidal or biphasic alternating current, modulates endogenous brain oscillations, and induces changes in synaptic plasticity to improve long-term brain function and depressive symptoms [67,68,69]. tACS uses different parameter stimulation electrodes, alternating current to stimulate specific targets. There are no acknowledged parameters, including stimulation frequency, intensity, phase, and duration, which may influence the effects of tACS. The selection of targets is based on the pathophysiological basis of the disease, with the frontal lobe being frequently used. The commonly used frequencies are mainly θ (4 to 7 Hz), α (8 to 14 Hz), β (14 to 30 Hz), and γ (30 to 80 Hz) bands [70,71]. The θ-band is associated with working memory and situational memory [70]. The α-band is strongly associated with executive function and attention [72]. The β-band is associated with motor ability, executive power, attention, and working memory [73]. The γ-band is associated with information processing, working memory, and situational memory [74]. The sensitivity of neurons varies among different intensities of tACS. Very-low-intensity tACS (<0.4 mA) acts preferentially on inhibitory neurons and activates inhibitory circuits; low-intensity tACS (0.6–0.8 mA) may activate inhibitory and excitatory neurons, which interact and cancel each other out, leading to no significant change in cortical excitability; higher-intensity tACS (>2 mA) activates the cortical excitatory circuit [75]. However, a higher intensity of tACS increases the risk of adverse effects such as headache and skin sensitization. Therefore, most tACS studies used alternating currents with peaks of no more than 4 mA [76]. There is no standard treatment duration for tACS. The durations used in previous studies ranged from 5 to 40 min. Most clinical trials have shown that tACS produces after effects (effects that persist after discontinuation of stimulation) in the 10–30 min range [77]. The most commonly used duration is a single 20 min treatment session once a day for 3 weeks, followed by a treatment session every other day or as needed [78]. However, when combining tACS with magnetoencephalography, one should prevent short-circuiting adjacent electrodes due to conductive fluids between electrodes [79].

As a new transcranial electrical stimulation technique, tACS can be considered a variation of the traditional tDCS [80]. It generates rhythmic currents at specific frequencies to induce specific frequencies of neural oscillations, thereby modulating the frequency and amplitude of cortical background oscillations to externally applied oscillations. Then, it modulates the synaptic plasticity or neurophysiological activity in specific brain regions and neural circuits between brain regions in a noninvasive, safe, and modifiable manner. Multicenter double-blind clinical trials with large sample size are needed to verify the reproducibility and representativeness of the results, to screen for optimal tACS stimulation parameters and to exclude parameters with adverse effects in order to obtain just the right therapeutic effect. It has also been suggested that the optimal tACS parameters may not be fixed. Combining tACS with techniques such as EEG and fMRI can provide individualized and adaptive stimulation parameters using feedback from the electrophysiological brain signals, which may help to improve the effectiveness of tACS and advance the application of tACS in clinical practice [81,82,83].

#### 3.1.3. tFUS

Ultrasound is an acoustic wave with a frequency greater than the hearing detection level of the human ear (>20 kHz); it is a mechanical wave generated by the vibration of a sound source and propagated through a compressed and expanded medium. tFUS, especially MR-guided focused ultrasound (MRgFUS) [16], combines the properties of deep focus targeting with high spatial resolution. It can stimulate deep and relatively superficial brain tissue in a precise, stable, focused, and noninvasive manner [84], and it has become one of the hot spots in basic and clinical research. The neuromodulatory effect of tFUS is mainly produced by the mechanical, thermal, and cavitation effects of ultrasound. By changing the frequency, intensity, pulse repetition frequency, pulse width, and duration of the ultrasound, the neurons at the stimulation site are activated or inhibited, thus regulating the neural function [17].

There are no clear criteria for the classification of tFUS, and the safety threshold for adult diagnosis defined in FDA guidelines is also used for adult cephalic ultrasound and neuromodulation. Its parameters include intensity spatial-peak pulse-average (Isspa) ≤ 190 W/cm^2^, intensity spatial-peak temporal-average (Ispta) ≤ 94 mW/cm^2^, and a mechanical index ≤ 1.9 [17]. Matt et al. demonstrated for the first time that low-intensity tFUS on S1 increased the efficiency of signal transmission in the sensorimotor network and improved white-matter microstructure in the stimulated regions [85]. Subsequently, many reports have investigated different parameters of tFUS in terms of frequency, intensity, and duration in order to obtain better treatment effects.

The inhibitory neuromodulation of tFUS is mainly achieved by high-intensity focused ultrasound (>1 W/cm^2^), which can cause a decrease in axonal action potential amplitude and conduction velocity or disrupt the ultrastructure of synapses and inter-synaptic connections of neurons in the brain through thermal effects. It, therefore, inhibits neuronal electrical activity and temporarily or permanently blocks nerve conduction [86]. It is commonly used to treat irreversible lesions [87,88]. In contrast, the activating effect is produced by low-intensity tFUS (≤1 W/cm^2^), which acts on the phospholipid bilayer of neuronal cells through mechanical and cavitation effects, affecting sodium, potassium, and calcium channels, and promoting the opening of these voltage-gated ion channels. Accordingly, it can promote action potentials, increase neuronal cell membrane permeability by generating nanobubbles in the phospholipid bilayer, and promote interneuronal synaptic transmission [89,90], ultimately selectively stimulating brain local activity and modulating the excitability of neural circuits [91,92].

Low-frequency ultrasound (250–350 kHz) is more effective than high-frequency (500–650 kHz) for neuromodulation [17,91]. The frequency and duration of pulse repetition and duty cycle can influence the effect generated by tFUS, but a unified agreement is lacking. Pulsed ultrasound modulation with low frequency, low intensity, 50% duty cycle, and 300 ms duration is generally considered to be most effective [17].

#### 3.1.4. tVNS

tVNS is a neuromodulation technique that involves applying electrical stimulation on peripheral vagus nerves. It transmits signals to the brain, causing changes in brain electrical activity and neurotransmitters, thereby modulating the functional activity of neurons [93]. tVNS can be mainly classified into two types: one is transcutaneous auricular vagus nerve stimulation (taVNS), which consists of electrically stimulating the auricular branch of the vagus nerve [94] (especially the auricular vessels); and the other is transcutaneous cervical vagus nerve stimulation (tcVNS), which consists of electrically stimulating the vagus nerve in the carotid sheath of the anterolateral neck [95]. 

tVNS exerts neuromodulatory effects, mainly by transmitting electrical stimulation to the brain, as well as increasing the plasticity of neural activity in the left prefrontal cortex, motor cortex, sensory cortex [96], right caudate nucleus, middle cingulate gyrus, and cerebellum [97]. It also enhances the plasticity of corticospinal motor pathways by activating widely projected neuromodulatory systems [98], ultimately increasing synaptic connections to muscle tissue and enhancing motor function [99].

Morrison et al. [100] showed that vagal stimulation at a moderate intensity of 0.8 mA enhanced motor cortex plasticity well, unlike vagal stimulation at a low intensity of 0.4 mA and a high intensity of 1.6 mA. Furthermore, an animal experiment [101] demonstrated that tVNS at a medium intensity of 0.8 mA significantly improved functional recovery compared with vagal stimulation at 0.4 mA or 1.6 mA intensity. An inverted U-shaped relationship between the degree of cortical plasticity induced by tVNS and its frequency has been found, with vagal stimulation at moderate frequencies (30 Hz) enhancing cortical plasticity, whereas neither slower (7.5 Hz) nor faster (120 Hz) pulse frequencies significantly enhance plasticity [102]. Therefore, tVNS with frequencies of 20 Hz, 25 Hz, and 30 Hz are commonly used in clinical trials for post-stroke rehabilitation [19,103]. In addition, the enhancement of cortical plasticity and memory due to vagal stimulation is also influenced by pulse width. Increasing pulse width can compensate for the decrease in stimulus intensity [104]. tVNS can reduce neurological deficits and has good efficacy in combination with rehabilitation in improving muscle function in patients with chronic stroke. However, more basic and clinical studies are needed to fully understand the mechanisms of efficacy, especially with large samples of phase III trials [105].

### 3.2. Application of NIBS in Post-Stroke Rehabilitation

The use of NIBS in clinical practice is continuously developing, and its role and position in neurological rehabilitation have become increasingly prominent. Combined with adjunctive exercises, the application of NIBS has been extrapolated from the treatment of post-stroke motor dysfunction to sensory disorders, aphasia, dysphagia, unilateral neglect, cognitive dysfunction, depression, and even disorders of consciousness in the acute phase [106].

#### 3.2.1. Motor Dysfunction

The most widely used application of NIBS in post-stroke rehabilitation is the treatment for motor dysfunction, especially rTMS and tDCS. In general, LF-rTMS (<1 Hz) decreases the excitability of the motor cortex, while HF-rTMS (≥5 Hz) activates the cortical excitability and increases the amplitude of motor-evoked potential (MEP) related to motor function improvement. In a randomized controlled trial conducted by Du et al. [107] in patients with early stroke, cortical excitability and exercise-induced fMRI activation in the ipsilateral motor area were significantly increased after HF-rTMS, whereas they were significantly decreased in the contralateral motor cortex after LF-rTMS. Patients were found to have an increase in Fugl-Meyer scores in both strategies after treatment and 3-month follow-up, suggesting that rTMS could improve motor function at the early post-stroke stage, which was related to motor cortical activation. Combining rTMS with hand grip and treadmill training would significantly improve hand motor coordination, motor speed, hand grip strength, walking speed, and spatial asymmetry, and quality of life in post-stroke patients [107,108,109]. Many studies have reported that rTMS can achieve modulation of the contralateral M1, supplementary motor areas, thalamus, postcentral gyrus, and frontal lobe, as well as other multi-brain areas through inter-region connections, in addition to intervention in the affected M1 area [110,111,112]. Current investigations have focused on the possible mechanism via which rTMS may enhance its functional connectivity with another brain region by modulating the neural circuit in which it is located through single-target stimulation [113,114].

tDCS is another common neuromodulation technique applied for motor dysfunction in post-stroke patients. In general, it is considered to generate similar effects of rTMS on improving limb motor function in stroke patients by stimulating the M1 cortex with anodal electrodes [115]. Bolognini et al. [1] found that hand grip strength, motor power index, and Barthel index were improved after treatment of tDCS over bilateral motor cortices for a 5-day session in post-stroke patients suffering from severe motor impairment. This suggested that tDCS accelerates motor recovery of the upper limb. It has been shown that stimulation of specific motor areas can selectively improve proximal or distal motor function of the upper limb [116]. Ojardias et al. [117] found that when tDCS was applied to M1 of the affected lower limb in patients with chronic hemiplegia, there was a significant increase in the patient’s distance in a 6 min walk test after a single treatment session. The patient’s overall walking ability also improved significantly. As a variant and innovative form of tDCS, tACS is still seldom used, although it is reportedly efficient in improving motor function. tACS oscillatively re-establishes connections between brain regions [118], promotes motor recovery, and improves cerebral hemodynamics after stroke [119]. There are still limitations concerning the effects of tDCS on improving motor function in post-stroke patients. Straudi et al. [120] reported that tDCS combined with robotics may be more beneficial for chronic and subcortical stroke patients than acute and cortical ones. As ineffectiveness has also been reported, many investigators choose to conduct tDCS combined with movement training or other rehabilitative therapies to achieve better outcomes. One study [121] compared tDCS with tACS or tRNS, and found that tRNS was more effective than tACS in increasing cortical excitability and may be the most effective tES method for stroke rehabilitation, with both being superior to tDCS. It is suggested that to obtain the maximum benefit of post-stroke rehabilitation, the future direction of tES should be more focused on high-definition stimulation [122], selecting for more effective stimulation parameters (frequency, intensity, and time) [123,124], combined with other rehabilitation modalities [125] and stimulation with synchronized functional images or EEG [126].

tFUS has become an emerging and attractive NIBS technique due to its high spatial resolution and deeper target of brain stimulation [127]. Although many animal trials have reported the benefits of tFUS for stroke [128,129,130,131], its application in post-stroke patients is still rare. We can identify from the limited number of studies of tFUS in humans that it can stimulate M1 motor areas with high spatial precision, thus modulating fine hand activity [132]. The mechanism may be related to the fact that low-intensity tFUS can noninvasively affect human brain activity by inhibiting cortical-evoked potentials, affecting cortical oscillatory dynamics, and altering the outcome of motor tasks [133].

The literature has shown that tVNS has the potential to enhance central noradrenergic activity and improve clinical outcomes in stroke. Studies from Capone et al. [134] and Wu et al. [105] demonstrated that tVNS combined with robotic rehabilitation improved upper-limb motor function and increased the ability of daily life in post-stroke patients. Other similar studies have shown that tVNS combined with rehabilitation training [135] is a safe and feasible treatment with good time dependence effects [136] and can be an effective assistive neurorehabilitation technique [137]. A multicenter, triple-blind, randomized controlled trial involving 108 patients conducted at 19 rehabilitation facilities in the U.K. and USA also confirmed that tVNS (18 times in 6 weeks) combined with rehabilitation was a safe and effective treatment for patients with long-term moderate to severe arm injury after ischemic stroke [138].

#### 3.2.2. Sensory Dysfunction

Sensory signals impact motor function by feeding information about the external environment and the intrinsic physiological state, as well as by directing the activation of the motor system. The impairment of sensory function after stroke, which injures motor control, would prevent recovery of motor function and reduce quality of life. Motor and sensory rehabilitation reinforce each other in a positive feedback loop [139]. Current studies on the effects of NIBS on superficial sensation in stroke patients have yielded some positive results, especially for tactile sensation. Baig et al. [19] performed taVNS for 6 weeks in 12 patients with ischemic stroke and found improvement in light tactile sensation after treatment. Another study also reported that 20 sessions of 2 h of VNS treatment combined with tactile sensation resulted in significant improvements in sensory function [140]. In addition, tRNS and anodal tPCS can improve tactile discrimination by modulating neuronal activity in the primary somatosensory cortex [141]. In contrast, the effect of tDCS on superficial sensation in post-stroke patients is mainly reflected in nociception and temperature perception [142].

In recent years, there has been an increasing number of studies on the effects of NIBS on deep sensation in post-stroke patients. Li et al. [143] found a significant trend of improvement in Fugl-Meyer somatosensory scores in acute stroke patients after taVNS combined with conventional rehabilitation training at 1-year follow-up, suggesting that taVNS can improve sensory function in post-stroke patients [19]. There was a significant increase in hand tactile thresholds and two-point discrimination sensation after HF-rTMS was applied to post-stroke patients, suggesting that HF-rTMS improves hand sensory function in chronic stroke patients, but no improvement was found in vibration, kinesthetic, or proprioceptive sensation [144]. In contrast, Koo et al. [145] found that tDCS showed significant improvements in deep sensation, tactile, pinprick, kinesthetic, and stereoscopic sensation in post-stroke patients. A trial using transcranial pulse stimulation (TPS) as a new low-intensity tFUS modality provided the first evidence [85] that tFUS improves functional connectivity between the primary somatosensory cortex and adjacent sensorimotor areas, which leads to improved sensory function and fine motor movements. Although the study was conducted in healthy participants, it provides ideas for the use of tFUS in post-stroke sensory dysfunction. It has also been shown that prolonged continuous theta burst stimulation (pcTBS) modulates the sensitivity of Aβ fibers and pain thresholds in healthy adults. Single pcTBS placed on the left M1 increases the excitability of DLPFC and FPC, suggesting that the interaction between M1 and PFC may be a potential mechanism for the analgesic effects of HF-rTMS [146]. However, studies in patients with central post-stroke pain are needed to confirm the clinical effects of pcTBS.

#### 3.2.3. Aphasia

Approximately one-third of stroke patients are diagnosed with aphasia [147], which exerts negative effects on functional outcomes, mood, quality of life, social participation, and the ability to return to work. Language impairment following post-stroke aphasia is heterogeneous, with many subtypes, including varying degrees of difficulty in comprehension, spontaneous speech, reading, or writing. Each subtype is associated with damage to specific cortical areas and may extend to subcortical areas. The use of NIBS techniques for aphasia rehabilitation has aroused wide interest, especially the application of TMS and tDCS. Growing evidence has verified their effectiveness in modulating cortical excitability [148], facilitating functional reorganization [149], and improving speech and language performance [150]. 

LF-rTMS was applied to the right-hemisphere regions to suppress cortex activation during language-related tasks and to encourage left-hemisphere activation around the lesion [151,152]. HF-rTMS, however, is used to promote activation of the remaining left hemisphere region [153] or the right hemisphere region [154]. Both LF- and HF-rTMS contribute to improved language outcomes in both subacute and chronic aphasia. However, the sample sizes of most studies are small, and only a few are randomized sham-controlled trials [152].

Most studies explored the effect of excitatory anodal stimulation of tDCS targeting the area around or ipsilateral to left-hemisphere lesion [155,156,157,158], with a few concentrating on inhibitory cathodal stimulation of healthy right hemisphere to suppress transhemispheric inhibition and thus greatly activate the impaired left hemisphere [159]. Some studies have focused on the application of tDCS in bilateral hemispheres, aiming to simultaneously combine anodal stimulation with cathodal stimulation to increase left hemisphere excitability and decrease right hemisphere excitability [160,161]. A Recent tDCS approach for aphasia targeted the right cerebellum [162], an area structurally and functionally connected to the language areas of the left hemisphere, which is particularly relevant with a large lesion in the left hemisphere, where it may be difficult to find live tissue to stimulate. The results of tDCS for aphasia were mostly positive, although the sample sizes of patients involved were small. Only two tDCS studies enrolled more than 50 patients [157,163]. Although it is not possible to determine an optimal method for improving speech function, both tDCS and TMS are prospective tools. However, due to being low-priced, portable, seizure risk-free [163], and easily paired with simultaneous speech therapy, tDCS is suitable for a wide range of clinical applications. Studies or clinical trials of other NIBS techniques in aphasia are not available and further research is expected.

#### 3.2.4. Dysphagia

The prevalence of oropharyngeal dysphagia after stroke is high [164], which is characterized by reduced swallowing efficiency and safety, leading to increased morbidity and mortality associated with nutritional and respiratory alterations. The neural circuits that generate swallowing patterns are located in the medulla oblongata [165], with extensive cortical and subcortical activation associated with motor preparation and sensory processing [166]. Thus, dysphagia or swallowing difficulties can be induced by stroke-related lesions in the cortical hemispheres, subcortical control circuits, or brainstem [167]. Evidence suggests that neurorepair and increased cortical activity play an important role in swallowing rehabilitation after stroke. NIBS, which is a particularly concerned treatment for post-stroke dysphagia [168], may promote cortical reorganization to accelerate the natural process of stroke rehabilitation.

Both HF- and LF-rTMS can be used for the treatment of post-stroke dysphagia, in which the commonly used frequencies are 10 Hz and 1 Hz [169]. The commonly used stimulation modes are bilateral high-frequency (10 HZ), ipsilateral high-frequency combined with contralateral low-frequency, and contralateral HF-rTMS stimulation of the pharyngeal motor cortex [169], in which bilateral high-frequency stimulation of the motor cortex is most effective for post-stroke dysphagia [170,171].

Anodal tDCS has also been used to improve swallowing function in post-stroke patients. A meta-analysis found a moderate but nonsignificant combined effect size for unilateral tDCS treatment compared with sham controls, although studies differed in terms of hemisphere stimulated, treatment regimen, and outcome measures [172]. Recent studies exploring bilateral tDCS have yielded positive results [173]. However, current studies vary considerably in terms of electrode placement site (ipsilateral versus contralateral), stimulation parameters (intensity and duration), stimulation pattern (unilateral versus bilateral), time after stroke, and the type of stroke patient who would benefit most. Further studies are needed to determine the optimal method of tDCS for the treatment of stroke patients with dysphagia.

Despite significant benefits in swallowing rehabilitation through the use of NIBS in most studies and their increasing acceptance by patients and clinical providers [174], it remains difficult to draw supportive conclusions about the efficacy of these neurostimulation techniques given the wide variation between studies [175], and further randomized controlled studies with standardized protocols are necessary to promote NIBS as a mainstream clinical treatment option for dysphagia [176].

#### 3.2.5. Cognitive Impairment

Post-stroke cognitive impairment (PSCI), which often occurs in survivors within 3 months after stroke [177], is mainly characterized by a decrease in memory, calculation, attention, and executive and reaction speed. rTMS and tDCS have been reported to improve the cognitive functional status in post-stroke patients by modulating the excitability of cortical circuits [178,179], which mainly manifests in small improvements of working memory, attention, and executive function [180,181]. Currently, NIBS usually produces modulation of cognitive functional circuits by bilateral or unilateral stimulation of the frontal lobe, especially the DLPFC [178]. Most studies included a small sample of patients, evaluated only short-term effects, and had too much variability in stimulation parameters; therefore, there is limited evidence to recommend rTMS [182] or tDCS [183,184] protocols for PSCI. In particular, the parameters of rTMS [182] varied in range from 1 Hz to 20 Hz in frequency, from 120 to 2000 in the total number of pulses, and from 1 to 20 in the total number of treatments. Future studies are needed to elucidate the appropriate stimulation targets, optimal parameters, and stimulation protocols to overcome individual differences [185] as well as obtain powerful evidence to support the application of NIBS in clinical practice [178].

#### 3.2.6. Unilateral Neglect

Unilateral neglect (UN) is a common functional impairment type after stroke and is negatively correlated with functional survival [186]; thus, its therapeutic intervention is an important part of stroke rehabilitation. NIBS, especially TBS [186], can be a promising rehabilitation option to favorably affect UN in post-stroke patients [18]. According to Cazzoli’s review, inhibitory rTMS seems to be a viable and effective approach to ameliorate neglect symptoms [187]. Studies have also reported that 1 Hz TMS stimulation on the posterior parietal cortex (P5) of the left hemisphere [188,189] with sensory cues significantly improved attention and hemiplegic upper-limb motor and daily living abilities in post-stroke patients with UN.

tDCS can also ameliorate UN after stroke by inhibiting excitability in the healthy hemisphere or by enhancing excitability in the affected hemisphere. The current study showed that cathodal tDCS (c-tDCS) eliminated the improvement of neglect after prism adaptation (PA), while anodal tDCS (a-tDCS) over the posterior parietal cortex (PPC) of the impaired hemisphere could promote the improvement of neglect after PA [190]. It has also been reported that the application of bimodal tDCS over bilateral PPC has a greater effect than single stimulation mode or sham stimulation mode. It is an effective treatment for unilateral visuospatial neglect in post-stroke patients [191]. Therefore, the method and specific effects of tDCS for UN due to stroke are still controversial, warranting further exploration of which stimulation modality is more effective [192]. Other NIBS techniques for UN after stroke are relatively rare. However, several reviews and meta-analyses have mentioned that TMS, especially the TBS model [186], is beneficial for the improvement of UN [193]. Existing evidence still cannot support or refute the NIBS technique [194]. Improving the quality of studies and obtaining more reliable evidence is the direction of future research [195].

#### 3.2.7. Post-Stroke Depression

Post-stroke depression (PSD) is the most common and burdensome neuropsychiatric complication after stroke [196]. The cross-sectional prevalence of PSD was estimated from 18% to 33% [197]. The pathophysiology of PSD is multifactorial [198] and probably involves reduced monoamine levels, abnormal neurotrophic responses, increased inflammation, and dysregulation of the hypothalamic–pituitary–adrenal axis, and glutamate-mediated excitotoxicity. These processes appear to be most relevant and obvious in the frontal lobes, hippocampus, limbic regions, and projections from the basal ganglia [199,200], especially DLPFC.

rTMS is the most frequently studied NIBS modality with few severe side effects. It can significantly improve PSD by modulating specific brain regions, such as activating the left DLPFC [201]. The effectiveness of rTMS applied in PSD has been reported in detail by several meta-analyses [201,202,203]. However, due to the limitations of failure to reach deep brain regions, as well as the large variability and small sample size of the included study protocols, further homogeneous randomized controlled trials with large samples are warranted to validate the long-term effects of rTMS.

Although tDCS is usually applied in treating depression [204], its effect on PSD has not been fully illustrated. Valiengo et al. [205] first demonstrated that tDCS can improve Hamilton Depression Rating Scale scores in patients with PSD through a randomized controlled trial. It is an effective intervention and may be one of the directions to be explored in the future. 

## 4. Current Status and Prospects

As the most commonly used neuromodulation technique, NIBS has achieved preliminary effects. Many clinical studies have reported the effectiveness of these techniques [28], but reports of the ineffectiveness of a single NIBS technique should not be neglected [206]. Therefore, the present paper reviews these findings and discusses future directions.

### 4.1. Effectiveness and Sustainability

Due to the heterogeneity of stroke injury location and the differences in the site, frequency, and duration of NIBS stimulation, it is difficult to use uniform criteria to judge the effectiveness and long-term efficacy of NIBS. However, there is a time-dependent effect of NIBS. In particular, cognitive function is significantly correlated with the number and duration of stimulation [182], which has been well-validated by recent studies. The session of commonly used treatments for NIBS reported in studies generally ranges from 5 to 20 (1–4 weeks), with follow-up usually after the end of treatment; the longest reported effective duration is 1 year [136].

### 4.2. Limitations and Future Trends

#### 4.2.1. Small Samples, and Insufficient and Short Follow-Up Studies

Firstly, most studies on the application of NIBS in stroke only included small sample sizes, usually fewer than 30 individuals in each group [207,208,209]. Due to the complexity of injury and the diversity of dysfunction, both of which have large individual variations, studies with limited sample sizes may result in less stable findings and fail to reliably reveal the true effects of noninvasive brain stimulation. It is necessary to carry out large-sample, multicenter, double-blind randomized controlled clinical trials in the future for the same treatment protocol of a single technology to further test the stability and reliability effects of NIBS.

Secondly, the current research on NIBS for stroke is still inadequate and insufficient. Specifically, most studies focused on motor function, speech, and cognitive function after stroke, mainly using rTMS and tDCS for intervention, while there are fewer studies investigating other functional impairments or using alternate NIBS techniques. At the same time, considering that complex neuromodulatory mechanisms may be involved in the dysfunction of stroke, the function of the brain may be based on the overall regulation of neural circuits or brain networks. Therefore, it is necessary to explore the altered function of stroke patients by integrating microscopic neuronal firing and neurotransmitter release, as well as macroscopic brain response signals and somatic nervous system signal changes at the level of the brain network or neural circuit for these NIBS techniques. Comparing sham stimulation groups for scale or behavioral assessment is also needed, so as to reveal the mechanism of regulation of neural circuits by each NIBS means in a more comprehensive way. 

Lastly, the follow-up duration after NIBS intervention in stroke patients was relatively short. Most studies assessed outcome indicators at the end of the intervention course, with the longest follow-up duration being 1 year [136]. It is necessary to extend the follow-up time to further clarify the effect of NIBS intervention in stroke.

#### 4.2.2. Variety of Therapeutic Options and Prospects

In previous studies, M1 and DLPFC were most frequently used as single stimulation targets, while other brain regions such as supplementary motor areas, S1, dACC, thalamus, and hippocampus have been rarely reported. Some recent studies found that functional connections are damaged in post-stroke patients [210]. Stimulating one or two targets of the same neural circuit can modulate the whole circuit and achieve better functional recovery. PAS protocols [211] including within-system, cross-system, and cortico–cortical, which are composed of rTMS or peripheral stimulation, provide the concept and feasibility of bi-targeted neuromodulation, especially cortico–cortical PAS [37,212]. On the basis of the understanding of brain network doctrine and the summary analysis of existing studies, we believe that neuromodulation can go from single-target stimulation to two-target stimulation, and then to multi-target stimulation of the same circuit, eventually achieving stimulation of the whole brain network, which will be a future development direction. Animal experiments have reported similar approaches, such as modulation of touch being able to improve dexterous motor function [213].

Secondly, the protocols of these neuromodulation techniques in NIBS applied in stroke patients still need further optimization and can be combined into exponentially increasing stimulation programs depending on the choice of stimulation location, intensity, frequency, total time, and whether the stimulation is continuous or not [214], among which the treatment parameters that may lead to adverse effects need further modification [206]. The optimal parameters for TMS, tES, tFUS, and tVNS need to be more clearly defined and harmonized, and more comprehensive well-designed high-quality studies on the selection of optimal parameters are expected to provide evidence for the early development of standardized therapeutic protocols for various techniques of NIBS. Additionally, some factors, such as contact with the operator or clinician, enable participants to generate specific expectations even before group allocation, which may further interfere with the effects of interventions and should be considered when implementing treatments [215].

Lastly, most of the available studies focused on examining the therapeutic effects and neuromodulatory mechanisms of a particular technique in stroke, but the possibility of combining different NIBS techniques in stroke is often ignored. The main reasons for this may be the difficulty in assessing the effects of combining various techniques and the difficulty in explaining the interactions between the combinations. Given the differences in the mechanisms of various NIBS, the integration of different techniques may enhance the neuromodulatory effect, and different NIBS combination models have been reported in the literature for application in stroke patients, such as rTMS-tDCS and TMS-tACS [216]. There are also many studies combining NIBS with fMRI or EEG [212] techniques, for example, TMS-EEG, and TMS-fMRI can be used in combination to better individualize and synchronize neuromodulation in stroke patients, revealing possible remote top-down effects at the neural population level [113]. Furthermore, the combination of cathodal cerebellar tDCS and visual feedback was reported to improve balance control in a healthy population [217] and these findings should also be considered to deeply elaborate the mechanism of NIBS techniques in post-stroke dysfunction, which will be a future direction of development.

## 5. Conclusions

In summary, a growing number of studies suggests that NIBS can be used as a therapeutic measure in post-stroke neurorehabilitation to promote recovery of functional impairment in patients when combined with movement training or other rehabilitation treatments. Overcoming the limitations of existing studies and using new stimulation modalities based on fMRI or EEG such as multitarget, bipolar, individualized, and multimodal stimulation, in which tDCS/tACS, tFUS, tVNS, and rTMS are combined with each other, NIBS can provide better and more precise modulation of neural circuits and neural networks, reduce adverse effects, and improve therapeutic effectiveness. This brings new opportunities for some patients with poor effect of conventional treatment, and strongly promotes its clinical application in the field of stroke neuromodulation.

## Data Availability

All reference cited in the article were collected from publicly available datasets.

## References

[1] Bolognini N., Russo C., Carneiro M.I.S., Nicotra A., Olgiati E., Spandri V., Agostoni E., Salmaggi A., Vallar G., Souza M.C. (2020). Bi-hemispheric transcranial direct current stimulation for upper-limb hemiparesis in acute stroke: A randomized, double-blind, sham-controlled trial. Eur. J. Neurol..

[2] Geed S., Feit P., Edwards D.F., Dromerick A.W. (2021). Why Are Stroke Rehabilitation Trial Recruitment Rates in Single Digits?. Front. Neurol..

[3] Kim C.-H., Chu H., Kang G.-H., Sung K.-K., Kang D.G., Lee H.S., Lee S. (2019). Difference in gait recovery rate of hemiparetic stroke patients according to paralyzed side. Medicine.

[4] Labberton A.S., Barra M., Rønning O.M., Thommessen B., Churilov L., Cadilhac D.A., Lynch E. (2019). Patient and service factors associated with referral and admission to inpatient rehabilitation after the acute phase of stroke in Australia and Norway. BMC Heal. Serv. Res..

[5] Sun H., He X., Tao X., Hou T., Chen M., He M., Liao H. (2020). The CD200/CD200R signaling pathway contributes to spontaneous functional recovery by enhancing synaptic plasticity after stroke. J. Neuroinflamm..

[6] Wiest R., Abela E., Missimer J., Schroth G., Hess C.W., Sturzenegger M., Wang D., Weder B., Federspiel A. (2014). Interhemispheric Cerebral Blood Flow Balance during Recovery of Motor Hand Function after Ischemic Stroke—A Longitudinal MRI Study Using Arterial Spin Labeling Perfusion. PLoS ONE.

[7] Rahayu U.B., Wibowo S., Setyopranoto I., Romli M.H. (2020). Effectiveness of physiotherapy interventions in brain plasticity, balance and functional ability in stroke survivors: A randomized controlled trial. NeuroRehabil..

[8] Zhu X., Wang H. (2022). Research progress on the generation of spasticity and the mechanism of physical factor therapy. Chin. J. Rehabil..

[9] Liu A., Qin Y. (2014). Physical factor therapy in stroke dysphagia. Neural Inj. Funct. Reconstr..

[10] Children’s Rehabilitation Committee of China Society of Rehabilitation Medicine, Children’s Cerebral Palsy Rehabilitation Committee of China Disabled Persons’ Rehabilitation Association, Children’s Rehabilitation Committee of Rehabilitation Physicians Branch of China Medical Association, Editorial Committee of China Cerebral Palsy Rehabilitation Guidelines (2022) (2022). Chinese rehabilitation guidelines for cerebral palsy (2022) part 4: Rehabilitation treatment(Ⅰ). Chin. J. Appl. Clin. Pediatr..

[11] Veerbeek J.M., van Wegen E., van Peppen R., van der Wees P.J., Hendriks E., Rietberg M., Kwakkel G. (2014). What Is the Evidence for Physical Therapy Poststroke? A Systematic Review and Meta-Analysis. PLoS ONE.

[12] Li Y., Li K., Feng R., Li Y., Li Y., Luo H., Yu Q. (2022). Mechanisms of Repetitive Transcranial Magnetic Stimulation on Post-stroke Depression: A Resting-State Functional Magnetic Resonance Imaging Study. Brain Topogr..

[13] Brunelin J., Adam O., Mondino M. (2022). Recent advances in noninvasive brain stimulation for schizophrenia. Curr. Opin. Psychiatry.

[14] Zhang W., Deng B., Xie F., Zhou H., Guo J.-F., Jiang H., Sim A., Tang B., Wang Q. (2022). Efficacy of repetitive transcranial magnetic stimulation in Parkinson’s disease: A systematic review and meta-analysis of randomised controlled trials. Eclinicalmedicine.

[15] Strube W., Bunse T., Malchow B., Hasan A. (2015). Efficacy and Interindividual Variability in Motor-Cortex Plasticity following Anodal tDCS and Paired-Associative Stimulation. Neural Plast..

[16] Hynynen K., Jones R.M. (2016). Image-guided ultrasound phased arrays are a disruptive technology for non-invasive therapy. Phys. Med. Biol..

[17] Kim H., Chiu A., Lee S.D., Fischer K., Yoo S.-S. (2014). Focused Ultrasound-mediated Non-invasive Brain Stimulation: Examination of Sonication Parameters. Brain Stimul..

[18] Cha H.G., Kim M.K. (2015). Effects of repetitive transcranial magnetic stimulation on arm function and decreasing unilateral spatial neglect in subacute stroke: A randomized controlled trial. Clin. Rehabil..

[19] Baig S.S., Falidas K., Laud P.J., Snowdon N., Farooq M.U., Ali A., Majid A., Redgrave J.N. (2019). Transcutaneous Auricular Vagus Nerve Stimulation with Upper Limb Repetitive Task Practice May Improve Sensory Recovery in Chronic Stroke. J. Stroke Cerebrovasc. Dis..

[20] Di Pino G., Pellegrino G., Assenza G., Capone F., Ferreri F., Formica D., Ranieri F., Tombini M., Ziemann U., Rothwell J.C. (2014). Modulation of brain plasticity in stroke: A novel model for neurorehabilitation. Nat. Rev. Neurol..

[21] Lefaucheur J.-P., Antal A., Ayache S.S., Benninger D.H., Brunelin J., Cogiamanian F., Cotelli M., De Ridder D., Ferrucci R., Langguth B. (2017). Evidence-based guidelines on the therapeutic use of transcranial direct current stimulation (tDCS). Clin. Neurophysiol..

[22] Lefaucheur J.-P., Aleman A., Baeken C., Benninger D.H., Brunelin J., Di Lazzaro V., Filipović S.R., Grefkes C., Hasan A., Hummel F.C. (2020). Evidence-based guidelines on the therapeutic use of repetitive transcranial magnetic stimulation (rTMS): An update (2014–2018). Clin. Neurophysiol..

[23] Lee D.J., Elias G.J., Lozano A.M. (2018). Neuromodulation for the treatment of eating disorders and obesity. Ther. Adv. Psychopharmacol..

[24] Dan X., Haihua X., Hao L.I., Hong Z., Jie T., Ning Z. (2023). Effect of Different Modalities of Repetitive Transcranial Magnetic Stimulation on Post-stroke Upper Limb Motor Dysfunction: A Network Meta-analysis. Chin. Gen. Pract..

[25] Aparício L.V., Guarienti F., Razza L.B., Carvalho A.F., Fregni F., Brunoni A.R. (2016). A Systematic Review on the Acceptability and Tolerability of Transcranial Direct Current Stimulation Treatment in Neuropsychiatry Trials. Brain Stimul..

[26] Boonzaier J., Van Tilborg G.A.F., Neggers S.F.W., Dijkhuizen R.M. (2018). Noninvasive Brain Stimulation to Enhance Functional Recovery After Stroke: Studies in Animal Models. Neurorehabilit. Neural Repair.

[27] Comino-Suárez N., Moreno J.C., Gómez-Soriano J., Megía-García A., Serrano-Muñoz D., Taylor J., Alcobendas-Maestro M., Gil-Agudo A., Del-Ama A.J., Avendaño-Coy J. (2021). Transcranial direct current stimulation combined with robotic therapy for upper and lower limb function after stroke: A systematic review and meta-analysis of randomized control trials. J. Neuroeng. Rehabil..

[28] D’Anci K.E., Uhl S., Oristaglio J., Sullivan N., Tsou A.Y. (2019). Treatments for Poststroke Motor Deficits and Mood Disorders: A Systematic Review for the 2019 U.S. Department of Veterans Affairs and U.S. Department of Defense Guidelines for Stroke Rehabilitation. Ann. Intern. Med..

[29] Fregni F., El-Hagrassy M.M., Pacheco-Barrios K., Carvalho S., Leite J., Simis M., Brunelin J., Nakamura-Palacios E.M., Marangolo P., Venkatasubramanian G. (2021). Evidence-Based Guidelines and Secondary Meta-Analysis for the Use of Transcranial Direct Current Stimulation in Neurological and Psychiatric Disorders. Int. J. Neuropsychopharmacol..

[30] Navarro-López V., Molina-Rueda F., Jiménez-Jiménez S., Alguacil-Diego I., Carratalá-Tejada M. (2021). Effects of Transcranial Direct Current Stimulation Combined with Physiotherapy on Gait Pattern, Balance, and Functionality in Stroke Patients. A Systematic Review. Diagnostics.

[31] Pastore-Wapp M., Nyffeler T., Nef T., Bohlhalter S., Vanbellingen T. (2021). Non-invasive brain stimulation in limb praxis and apraxia: A scoping review in healthy subjects and patients with stroke. Cortex.

[32] Shen Q.-R., Hu M.-T., Feng W., Li K.-P., Wang W. (2022). Narrative Review of Noninvasive Brain Stimulation in Stroke Rehabilitation. Experiment.

[33] Gatzinsky K., Bergh C., Liljegren A., Silander H., Samuelsson J., Svanberg T., Samuelsson O. (2021). Repetitive transcranial magnetic stimulation of the primary motor cortex in management of chronic neuropathic pain: A systematic review. Scand. J. Pain.

[34] Jing Y., Zhao N., Deng X.P., Feng Z.J., Huang G.F., Meng M., Zang Y.F., Wang J. (2020). Pregenual or subgenual anterior cingulate cortex as potential effective region for brain stimulation of depression. Brain Behav..

[35] Timäus C., Vogelgsang J., Kis B., Radenbach K., Wolff-Menzler C., Mavridou K., Gyßer S., Hessmann P., Wiltfang J. (2021). Current clinical practice of electroconvulsive therapy and repetitive transcranial magnetic stimulation in psychiatry, a German sample. Eur. Arch. Psychiatry Clin. Neurosci..

[36] Wang Y., Cao N., Lin Y., Chen R., Zhang J. (2020). Hemispheric Differences in Functional Interactions Between the Dorsal Lateral Prefrontal Cortex and Ipsilateral Motor Cortex. Front. Hum. Neurosci..

[37] Guidali G., Roncoroni C., Bolognini N. (2021). Paired associative stimulations: Novel tools for interacting with sensory and motor cortical plasticity. Behav. Brain Res..

[38] Beynel L., Appelbaum L.G., Luber B., Crowell C.A., Hilbig S.A., Lim W., Nguyen D., Chrapliwy N.A., Davis S.W., Cabeza R. (2019). Effects of online repetitive transcranial magnetic stimulation (rTMS) on cognitive processing: A meta-analysis and recommendations for future studies. Neurosci. Biobehav. Rev..

[39] Schramm S., Haddad A., Chyall L., Krieg S., Sollmann N., Tarapore P. (2020). Navigated TMS in the ICU: Introducing Motor Mapping to the Critical Care Setting. Brain Sci..

[40] Lefaucheur J.-P., Ayache S., Sorel M., Farhat W., Zouari H., de Andrade D.C., Ahdab R., Ménard-Lefaucheur I., Brugières P., Goujon C. (2012). Analgesic effects of repetitive transcranial magnetic stimulation of the motor cortex in neuropathic pain: Influence of theta burst stimulation priming. Eur. J. Pain.

[41] Assogna M., Casula E.P., Borghi I., Bonnì S., Samà D., Motta C., Di Lorenzo F., D’Acunto A., Porrazzini F., Minei M. (2020). Effects of Palmitoylethanolamide Combined with Luteoline on Frontal Lobe Functions, High Frequency Oscillations, and GABAergic Transmission in Patients with Frontotemporal Dementia. J. Alzheimer’s Dis..

[42] Kim J.-H., Han J.-Y., Song M.-K., Park G.-C., Lee J.-S. (2020). Synergistic Effects of Scalp Acupuncture and Repetitive Transcranial Magnetic Stimulation on Cerebral Infarction: A Randomized Controlled Pilot Trial. Brain Sci..

[43] Solomon E.A., Sperling M.R., Sharan A.D., Wanda P.A., Levy D.F., Lyalenko A., Pedisich I., Rizzuto D.S., Kahana M.J. (2021). Theta-burst stimulation entrains frequency-specific oscillatory responses. Brain Stimul..

[44] Suppa A., Quartarone A., Siebner H., Chen R., Di Lazzaro V., Del Giudice P., Paulus W., Rothwell J.C., Ziemann U., Classen J. (2017). The associative brain at work: Evidence from paired associative stimulation studies in humans. Clin. Neurophysiol..

[45] Guse B., Falkai P., Wobrock T. (2010). Cognitive effects of high-frequency repetitive transcranial magnetic stimulation: A systematic review. J. Neural Transm..

[46] Peng Z., Zhou C., Xue S., Bai J., Yu S., Li X., Wang H., Tan Q. (2018). Mechanism of Repetitive Transcranial Magnetic Stimulation for Depression. Shanghai Arch Psychiatry.

[47] Groppa S., Oliviero A., Eisen A., Quartarone A., Cohen L., Mall V., Kaelin-Lang A., Mima T., Rossi S., Thickbroom G. (2012). A practical guide to diagnostic transcranial magnetic stimulation: Report of an IFCN committee. Clin. Neurophysiol..

[48] Chen R. (2020). Guideline on therapeutic use of repetitive transcranial magnetic stimulation: Useful but know the methods and limitations. Clin. Neurophysiol..

[49] Giustiniani A., Vallesi A., Oliveri M., Tarantino V., Ambrosini E., Bortoletto M., Masina F., Busan P., Siebner H., Fadiga L. (2022). A questionnaire to collect unintended effects of transcranial magnetic stimulation: A consensus based approach. Clin. Neurophysiol..

[50] Liu A., Vöröslakos M., Kronberg G., Henin S., Krause M.R., Huang Y., Opitz A., Mehta A., Pack C.C., Krekelberg B. (2018). Immediate neurophysiological effects of transcranial electrical stimulation. Nat. Commun..

[51] Thibaut A., Russo C., Morales-Quezada L., Hurtado-Puerto A., Deitos A., Freedman S., Carvalho S., Fregni F. (2017). Neural signature of tDCS, tPCS and their combination: Comparing the effects on neural plasticity. Neurosci. Lett..

[52] Peña J., Sampedro A., Ibarretxe-Bilbao N., Zubiaurre-Elorza L., Ojeda N. (2019). Improvement in creativity after transcranial random noise stimulation (tRNS) over the left dorsolateral prefrontal cortex. Sci. Rep..

[53] Nitsche M.A., Paulus W. (2000). Excitability changes induced in the human motor cortex by weak transcranial direct current stimulation. J. Physiol..

[54] Ebonsi P., Ecuomo D., Emartella G., Emadeo G., Eschirinzi T., Epuglisi F., Eponterio G., Episani A. (2011). Centrality of Striatal Cholinergic Transmission in Basal Ganglia Function. Front. Neuroanat..

[55] Goff D.C. (2017). D-cycloserine in Schizophrenia: New Strategies for Improving Clinical Outcomes by Enhancing Plasticity. Curr. Neuropharmacol..

[56] Li L.M., Uehara K., Hanakawa T. (2015). The contribution of interindividual factors to variability of response in transcranial direct current stimulation studies. Front. Cell. Neurosci..

[57] Hsu G., Farahani F., Parra L.C. (2021). Cutaneous sensation of electrical stimulation waveforms. Brain Stimul..

[58] Groppa S., Bergmann T., Siems C., Mölle M., Marshall L., Siebner H. (2010). Slow-oscillatory transcranial direct current stimulation can induce bidirectional shifts in motor cortical excitability in awake humans. Neuroscience.

[59] Woods A.J., Antal A., Bikson M., Boggio P.S., Brunoni A.R., Celnik P., Cohen L.G., Fregni F., Herrmann C.S., Kappenman E.S. (2016). A technical guide to tDCS, and related non-invasive brain stimulation tools. Clin. Neurophysiol..

[60] Coffman B.A., Clark V.P., Parasuraman R. (2014). Battery powered thought: Enhancement of attention, learning, and memory in healthy adults using transcranial direct current stimulation. Neuroimage.

[61] Shinde A.B., Lerud K.D., Munsch F., Alsop D.C., Schlaug G. (2021). Effects of tDCS dose and electrode montage on regional cerebral blood flow and motor behavior. Neuroimage.

[62] Hong-Yu L., Zhi-Jie Z., Juan L., Ting X., Wei-Chun H., Ning Z. (2022). Effects of Cerebellar Transcranial Direct Current Stimulation in Patients with Stroke: A Systematic Review. Cerebellum.

[63] Nitsche M.A., Paulus W. (2001). Sustained excitability elevations induced by transcranial DC motor cortex stimulation in humans. Neurology.

[64] Wagner T., Fregni F., Fecteau S., Grodzinsky A., Zahn M., Pascual-Leone A. (2007). Transcranial direct current stimulation: A computer-based human model study. Neuroimage.

[65] Zaehle T., Rach S., Herrmann C.S. (2010). Transcranial Alternating Current Stimulation Enhances Individual Alpha Activity in Human EEG. PLoS ONE.

[66] Kar K., Ito T., Cole M.W., Krekelberg B., Meehan C.E., Spooner R.K., Jones K.T., Johnson E.L., Tauxe Z.S., Rojas D.C. (2020). Transcranial alternating current stimulation attenuates BOLD adaptation and increases functional connectivity. J. Neurophysiol..

[67] Elyamany O., Leicht G., Herrmann C.S., Mulert C. (2021). Transcranial alternating current stimulation (tACS): From basic mechanisms towards first applications in psychiatry. Eur. Arch. Psychiatry Clin. Neurosci..

[68] Tavakoli A.V., Yun K. (2017). Transcranial Alternating Current Stimulation (tACS) Mechanisms and Protocols. Front. Cell. Neurosci..

[69] Vosskuhl J., Strüber D., Herrmann C. (2015). Transcranial alternating current stimulation. Entrainment and function control of neuronal networks. Nervenarzt.

[70] Mansouri F., Shanbour A., Mazza F., Fettes P., Zariffa J., Downar J. (2019). Effect of Theta Transcranial Alternating Current Stimulation and Phase-Locked Transcranial Pulsed Current Stimulation on Learning and Cognitive Control. Front. Neurosci..

[71] Zhang Y., Zhang Y., Cai P., Luo H., Fang F. (2019). The causal role of α-oscillations in feature binding. Proc. Natl. Acad. Sci. USA.

[72] Rioult-Pedotti M.-S., Friedman D., Donoghue J.P. (2000). Learning-Induced LTP in Neocortex. Science.

[73] Wischnewski M., Joergensen M.L., Compen B., Schutter D.J.L.G. (2020). Frontal Beta Transcranial Alternating Current Stimulation Improves Reversal Learning. Cereb. Cortex.

[74] Haller N., Senner F., Brunoni A.R., Padberg F., Palm U. (2020). Gamma transcranial alternating current stimulation improves mood and cognition in patients with major depression. J. Psychiatr. Res..

[75] Moliadze V., Atalay D., Antal A., Paulus W. (2012). Close to threshold transcranial electrical stimulation preferentially activates inhibitory networks before switching to excitation with higher intensities. Brain Stimul..

[76] Hohn V.D., May E.S., Ploner M. (2019). From correlation towards causality: Modulating brain rhythms of pain using transcranial alternating current stimulation. PAIN Rep..

[77] Strüber D., Rach S., Neuling T., Herrmann C.S. (2015). On the possible role of stimulation duration for after-effects of transcranial alternating current stimulation. Front. Cell. Neurosci..

[78] Kavirajan H.C., Lueck K., Chuang K. (2014). Alternating current cranial electrotherapy stimulation (CES) for depression. Cochrane Database Syst. Rev..

[79] Antal A., Alekseichuk I., Bikson M., Brockmöller J., Brunoni A., Chen R., Cohen L., Dowthwaite G., Ellrich J., Flöel A. (2017). Low intensity transcranial electric stimulation: Safety, ethical, legal regulatory and application guidelines. Clin. Neurophysiol..

[80] Röhner F., Breitling C., Rufener K.S., Heinze H.-J., Hinrichs H., Krauel K., Sweeney-Reed C.M. (2018). Modulation of Working Memory Using Transcranial Electrical Stimulation: A Direct Comparison Between TACS and TDCS. Front. Neurosci..

[81] Berger A., Pixa N.H., Steinberg F., Doppelmayr M. (2018). Brain Oscillatory and Hemodynamic Activity in a Bimanual Coordination Task Following Transcranial Alternating Current Stimulation (tACS): A Combined EEG-fNIRS Study. Front. Behav. Neurosci..

[82] Holzmann R., Koppehele-Gossel J., Voss U., Klimke A. (2021). Investigating Nuisance Effects Induced in EEG During tACS Application. Front. Hum. Neurosci..

[83] Indahlastari A., Kasinadhuni A.K., Saar C., Castellano K., Mousa B., Chauhan M., Mareci T.H., Sadleir R.J. (2018). Methods to Compare Predicted and Observed Phosphene Experience in tACS Subjects. Neural Plast..

[84] Kubanek J. (2018). Neuromodulation with transcranial focused ultrasound. Neurosurg. Focus.

[85] Matt E., Kaindl L., Tenk S., Egger A., Kolarova T., Karahasanović N., Amini A., Arslan A., Sariçiçek K., Weber A. (2022). First evidence of long-term effects of transcranial pulse stimulation (TPS) on the human brain. J. Transl. Med..

[86] Colucci V., Strichartz G., Jolesz F., Vykhodtseva N., Hynynen K. (2009). Focused Ultrasound Effects on Nerve Action Potential in vitro. Ultrasound Med. Biol..

[87] Fasano A., De Vloo P., Llinas M., Hlasny E., Kucharczyk W., Hamani C., Lozano A.M. (2018). Magnetic Resonance Imaging-Guided Focused Ultrasound Thalamotomy in Parkinson Tremor: Reoperation After Benefit Decay. Mov. Disord..

[88] Kim S.J., Roh D., Jung H.H., Chang W.S., Kim C.-H., Chang J.W. (2018). A study of novel bilateral thermal capsulotomy with focused ultrasound for treatment-refractory obsessive–compulsive disorder: 2-year follow-up. J. Psychiatry Neurosci..

[89] Kubanek J., Shi J., Marsh J., Chen D., Deng C., Cui J. (2016). Ultrasound modulates ion channel currents. Sci. Rep..

[90] Tyler W.J., Tufail Y., Finsterwald M., Tauchmann M.L., Olson E.J., Majestic C. (2008). Remote Excitation of Neuronal Circuits Using Low-Intensity, Low-Frequency Ultrasound. PLoS ONE.

[91] Tufail Y., Matyushov A., Baldwin N., Tauchmann M.L., Georges J., Yoshihiro A., Tillery S.I.H., Tyler W.J. (2010). Transcranial Pulsed Ultrasound Stimulates Intact Brain Circuits. Neuron.

[92] Yoo S.-S., Bystritsky A., Lee J.-H., Zhang Y., Fischer K., Min B.-K., McDannold N.J., Pascual-Leone A., Jolesz F.A. (2011). Focused ultrasound modulates region-specific brain activity. Neuroimage.

[93] Borges U., Knops L., Laborde S., Klatt S., Raab M. (2020). Transcutaneous Vagus Nerve Stimulation May Enhance Only Specific Aspects of the Core Executive Functions. A Randomized Crossover Trial. Front. Neurosci..

[94] Vosseler A., Zhao D., Fritsche L., Lehmann R., Kantartzis K., Small D.M., Peter A., Häring H.-U., Birkenfeld A.L., Fritsche A. (2020). No modulation of postprandial metabolism by transcutaneous auricular vagus nerve stimulation: A cross-over study in 15 healthy men. Sci. Rep..

[95] Wittbrodt M.T., Gurel N.Z., Nye J.A., Ladd S., Shandhi M.H., Huang M., Shah A.J., Pearce B.D., Alam Z.S., Rapaport M.H. (2020). Non-invasive vagal nerve stimulation decreases brain activity during trauma scripts. Brain Stimul..

[96] Takahashi H., Shiramatsu T.I., Hitsuyu R., Ibayashi K., Kawai K. (2020). Vagus nerve stimulation (VNS)-induced layer-specific modulation of evoked responses in the sensory cortex of rats. Sci. Rep..

[97] Badran B.W., Dowdle L.T., Mithoefer O.J., LaBate N.T., Coatsworth J., Brown J.C., DeVries W.H., Austelle C.W., McTeague L.M., George M.S. (2018). Neurophysiologic effects of transcutaneous auricular vagus nerve stimulation (taVNS) via electrical stimulation of the tragus: A concurrent taVNS/fMRI study and review. Brain Stimul..

[98] Collins L., Boddington L., Steffan P.J., McCormick D. (2021). Vagus nerve stimulation induces widespread cortical and behavioral activation. Curr. Biol..

[99] Meyers E.C., Solorzano B.R., James J., Ganzer P.D., Lai E.S., Rennaker R.L., Kilgard M.P., Hays S.A. (2018). Vagus Nerve Stimulation Enhances Stable Plasticity and Generalization of Stroke Recovery. Stroke.

[100] Morrison R.A., Hulsey D.R., Adcock K.S., Rennaker R.L., Kilgard M.P., Hays S.A. (2019). Vagus nerve stimulation intensity influences motor cortex plasticity. Brain Stimul..

[101] Pruitt D.T., Danaphongse T.T., Lutchman M., Patel N., Reddy P., Wang V., Parashar A., Rennaker R.L., Kilgard M.P., Hays S.A. (2021). Optimizing Dosing of Vagus Nerve Stimulation for Stroke Recovery. Transl. Stroke Res..

[102] Buell E., Loerwald K., Engineer C., Borland M., Buell J., Kelly C., Khan I., Hays S., Kilgard M. (2018). Cortical map plasticity as a function of vagus nerve stimulation rate. Brain Stimul..

[103] Li L., Wang D., Pan H., Huang L., Sun X., He C., Wei Q. (2022). Non-invasive Vagus Nerve Stimulation in Cerebral Stroke: Current Status and Future Perspectives. Front. Neurosci..

[104] Loerwald K.W., Borland M.S., Rennaker R.L., Hays S.A., Kilgard M. (2018). The interaction of pulse width and current intensity on the extent of cortical plasticity evoked by vagus nerve stimulation. Brain Stimul..

[105] Wu D., Ma J., Zhang L., Wang S., Tan B., Jia G. (2020). Effect and Safety of Transcutaneous Auricular Vagus Nerve Stimulation on Recovery of Upper Limb Motor Function in Subacute Ischemic Stroke Patients: A Randomized Pilot Study. Neural Plast..

[106] Krewer C., Thibaut A. (2020). Non-invasive brain stimulation for treatment of severe disorders of consciousness in people with acquired brain injury. Cochrane Database Syst. Rev..

[107] Du J., Yang F., Hu J., Hu J., Xu Q., Cong N., Zhang Q., Liu L., Mantini D., Zhang Z. (2019). Effects of high- and low-frequency repetitive transcranial magnetic stimulation on motor recovery in early stroke patients: Evidence from a randomized controlled trial with clinical, neurophysiological and functional imaging assessments. NeuroImage Clin..

[108] Wang R.-Y., Wang F.-Y., Huang S.-F., Yang Y.-R. (2019). High-frequency repetitive transcranial magnetic stimulation enhanced treadmill training effects on gait performance in individuals with chronic stroke: A double-blinded randomized controlled pilot trial. Gait Posture.

[109] Yang Y., Pan H., Pan W., Liu Y., Song X., Niu C.M., Feng W., Wang J., Xie Q. (2021). Repetitive Transcranial Magnetic Stimulation on the Affected Hemisphere Enhances Hand Functional Recovery in Subacute Adult Stroke Patients: A Randomized Trial. Front. Aging Neurosci..

[110] Diekhoff-Krebs S., Pool E.-M., Sarfeld A.-S., Rehme A.K., Eickhoff S.B., Fink G.R., Grefkes C. (2017). Interindividual differences in motor network connectivity and behavioral response to iTBS in stroke patients. NeuroImage Clin..

[111] Guo Z., Jin Y., Bai X., Jiang B., He L., McClure M.A., Mu Q. (2021). Distinction of High- and Low-Frequency Repetitive Transcranial Magnetic Stimulation on the Functional Reorganization of the Motor Network in Stroke Patients. Neural Plast..

[112] Li J., Zhang X.-W., Zuo Z.-T., Lu J., Meng C.-L., Fang H.-Y., Xue R., Fan Y., Guan Y.-Z., Zhang W.-H. (2016). Cerebral Functional Reorganization in Ischemic Stroke after Repetitive Transcranial Magnetic Stimulation: An fMRI Study. CNS Neurosci. Ther..

[113] Bestmann S., Ruff C.C., Blankenburg F., Weiskopf N., Driver J., Rothwell J.C. (2008). Mapping causal interregional influences with concurrent TMS–fMRI. Exp. Brain Res..

[114] Jin J.-N., Wang X., Li Y., Jin F., Liu Z.-P., Yin T. (2017). The Effects of rTMS Combined with Motor Training on Functional Connectivity in Alpha Frequency Band. Front. Behav. Neurosci..

[115] Kandel M., Beis J.-M., Le Chapelain L., Guesdon H., Paysant J. (2012). Non-invasive cerebral stimulation for the upper limb rehabilitation after stroke: A review. Ann. Phys. Rehabil. Med..

[116] Weightman M., Brittain J.-S., Punt D., Miall R.C., Jenkinson N. (2020). Targeted tDCS selectively improves motor adaptation with the proximal and distal upper limb. Brain Stimul..

[117] Ojardias E., Azé O.D., Luneau D., Mednieks J., Condemine A., Rimaud D., Chassagne F., Giraux P. (2020). The Effects of Anodal Transcranial Direct Current Stimulation on the Walking Performance of Chronic Hemiplegic Patients. Neuromodulation Technol. Neural Interface.

[118] Wu J.-F., Wang H.-J., Wu Y., Li F., Bai Y.-L., Zhang P.-Y., Chan C.C.H. (2016). Efficacy of transcranial alternating current stimulation over bilateral mastoids (tACS_bm_) on enhancing recovery of subacute post-stroke patients. Top. Stroke Rehabil..

[119] Takeuchi N., Izumi S.-I. (2021). Motor Learning Based on Oscillatory Brain Activity Using Transcranial Alternating Current Stimulation: A Review. Brain Sci..

[120] Straudi S., Fregni F., Martinuzzi C., Pavarelli C., Salvioli S., Basaglia N. (2016). tDCS and Robotics on Upper Limb Stroke Rehabilitation: Effect Modification by Stroke Duration and Type of Stroke. BioMed Res. Int..

[121] Inukai Y., Saito K., Sasaki R., Tsuiki S., Miyaguchi S., Kojima S., Masaki M., Otsuru N., Onishi H. (2016). Comparison of Three Non-Invasive Transcranial Electrical Stimulation Methods for Increasing Cortical Excitability. Front. Hum. Neurosci..

[122] Elsner B., Kugler J., Mehrholz J. (2018). Transcranial direct current stimulation (tDCS) for upper limb rehabilitation after stroke: Future directions. J. Neuroeng. Rehabil..

[123] Miyaguchi S., Otsuru N., Kojima S., Yokota H., Saito K., Inukai Y., Onishi H. (2019). Gamma tACS over M1 and cerebellar hemisphere improves motor performance in a phase-specific manner. Neurosci. Lett..

[124] Naros G., Gharabaghi A. (2017). Physiological and behavioral effects of β-tACS on brain self-regulation in chronic stroke. Brain Stimul..

[125] Shanmugasundaram V., Solomons C. (2019). A review of transcranial electrical stimulation methods in stroke rehabilitation. Neurol. India.

[126] Guleyupoglu B., Schestatsky P., Edwards D., Fregni F., Bikson M. (2013). Classification of methods in transcranial Electrical Stimulation (tES) and evolving strategy from historical approaches to contemporary innovations. J. Neurosci. Methods.

[127] Dell’Italia J., Sanguinetti J.L., Monti M.M., Bystritsky A., Reggente N. (2022). Current State of Potential Mechanisms Supporting Low Intensity Focused Ultrasound for Neuromodulation. Front. Hum. Neurosci..

[128] Guo T., Li H., Lv Y., Lu H., Niu J., Sun J., Yang G.-Y., Ren C., Tong S. (2015). Pulsed Transcranial Ultrasound Stimulation Immediately After The Ischemic Brain Injury is Neuroprotective. IEEE Trans. Biomed. Eng..

[129] Li H., Sun J., Zhang D., Omire-Mayor D., Lewin P.A., Tong S. (2017). Low-intensity (400 mW/cm^2^, 500 kHz) pulsed transcranial ultrasound preconditioning may mitigate focal cerebral ischemia in rats. Brain Stimul..

[130] Liu L., Du J., Zheng T., Hu S., Dong Y., Du D., Wu S., Wang X., Shi Q. (2019). Protective effect of low-intensity transcranial ultrasound stimulation after differing delay following an acute ischemic stroke. Brain Res. Bull..

[131] Wu S., Zheng T., Du J., Yuan Y., Shi Q., Wang Z., Liu D., Liu J., Wang X., Liu L. (2020). Neuroprotective effect of low-intensity transcranial ultrasound stimulation in endothelin-1-induced middle cerebral artery occlusion in rats. Brain Res. Bull..

[132] Ai L., Bansal P., Mueller J.K., Legon W. (2018). Effects of transcranial focused ultrasound on human primary motor cortex using 7T fMRI: A pilot study. BMC Neurosci..

[133] Fomenko A., Neudorfer C., Dallapiazza R.F., Kalia S.K., Lozano A.M. (2018). Low-intensity ultrasound neuromodulation: An overview of mechanisms and emerging human applications. Brain Stimul..

[134] Capone F., Miccinilli S., Pellegrino G., Zollo L., Simonetti D., Bressi F., Florio L., Ranieri F., Falato E., Di Santo A. (2017). Transcutaneous Vagus Nerve Stimulation Combined with Robotic Rehabilitation Improves Upper Limb Function after Stroke. Neural Plast..

[135] Engineer N.D., Kimberley T.J., Prudente C.N., Dawson J., Tarver W.B., Hays S.A. (2019). Targeted Vagus Nerve Stimulation for Rehabilitation After Stroke. Front. Neurosci..

[136] Dawson J., Engineer N.D., Prudente C.N., Pierce D., Francisco G., Yozbatiran N., Tarver W.B., Casavant R., Kline D.K., Cramer S.C. (2020). Vagus Nerve Stimulation Paired With Upper-Limb Rehabilitation After Stroke: One-Year Follow-up. Neurorehabil. Neural Repair.

[137] Dawson J., Pierce D., Dixit A., Kimberley T.J., Robertson M., Tarver B., Hilmi O., McLean J., Forbes K., Kilgard M.P. (2016). Safety, Feasibility, and Efficacy of Vagus Nerve Stimulation Paired With Upper-Limb Rehabilitation after Ischemic Stroke. Stroke.

[138] Dawson J., Liu C.Y., E Francisco G., Cramer S.C., Wolf S.L., Dixit A., Alexander J., Ali R., Brown B.L., Feng W. (2021). Vagus nerve stimulation paired with rehabilitation for upper limb motor function after ischaemic stroke (VNS-REHAB): A randomised, blinded, pivotal, device trial. Lancet.

[139] Pundik S., Scoco A., Skelly M., McCabe J.P., Daly J.J. (2018). Greater Cortical Thickness Is Associated With Enhanced Sensory Function After Arm Rehabilitation in Chronic Stroke. Neurorehabilit. Neural Repair.

[140] Kilgard M.P., Rennaker R.L., Alexander J., Dawson J. (2018). Vagus nerve stimulation paired with tactile training improved sensory function in a chronic stroke patient. NeuroRehabilitation.

[141] Saito K., Otsuru N., Inukai Y., Miyaguchi S., Yokota H., Kojima S., Sasaki R., Onishi H. (2019). Comparison of transcranial electrical stimulation regimens for effects on inhibitory circuit activity in primary somatosensory cortex and tactile spatial discrimination performance. Behav. Brain Res..

[142] Bae S.-H., Kim G.-D., Kim K.-Y. (2014). Analgesic Effect of Transcranial Direct Current Stimulation on Central Post-Stroke Pain. Tohoku J. Exp. Med..

[143] Niu L.-C., Li J.-N., Xie C.-C., Li C.-Q., Zhang G.-F., Tang H., Jin C.-N., Ma J.-X., Wen L., Zhang K.-M. (2022). Efficacy and safety of transcutaneous auricular vagus nerve stimulation combined with conventional rehabilitation training in acute stroke patients: A randomized controlled trial conducted for 1 year involving 60 patients. Neural Regen. Res..

[144] Pundik S., Skelly M., McCabe J., Akbari H., Tatsuoka C., Plow E.B. (2021). Does rTMS Targeting Contralesional S1 Enhance Upper Limb Somatosensory Function in Chronic Stroke? A Proof-of-Principle Study. Neurorehabilit. Neural Repair.

[145] Koo W.R., Jang B.H., Kim C.R. (2018). Effects of Anodal Transcranial Direct Current Stimulation on Somatosensory Recovery After Stroke. Am. J. Phys. Med. Rehabil..

[146] Li C., Zhang N., Han Q., Zhang L., Xu S., Tu S., Xie Y., Wang Z. (2022). Prolonged Continuous Theta Burst Stimulation Can Regulate Sensitivity on Aβ Fibers: An Functional Near-Infrared Spectroscopy Study. Front. Mol. Neurosci..

[147] Flowers H.L., Skoretz S.A., Silver F.L., Rochon E., Fang J., Flamand-Roze C., Martino R. (2016). Poststroke Aphasia Frequency, Recovery, and Outcomes: A Systematic Review and Meta-Analysis. Arch. Phys. Med. Rehabil..

[148] Szaflarski J.P., Vannest J., Wu S.W., DiFrancesco M.W., Banks C., Gilbert D.L. (2011). Excitatory repetitive transcranial magnetic stimulation induces improvements in chronic post-stroke aphasia. Med. Sci. Monit..

[149] van Oers C.A.M.M., van der Worp H.B., Kappelle L.J., Raemaekers M.A.H., Otte W.M., Dijkhuizen R.M. (2018). Etiology of language network changes during recovery of aphasia after stroke. Sci. Rep..

[150] Zumbansen A., Kneifel H., Lazzouni L., Ophey A., Black S.E., Chen J.L., Edwards D., Funck T., Hartmann A.E., Heiss W.-D. (2022). Differential Effects of Speech and Language Therapy and rTMS in Chronic Versus Subacute Post-stroke Aphasia: Results of the NORTHSTAR-CA Trial. Neurorehabilit. Neural Repair.

[151] Haghighi M., Mazdeh M., Ranjbar N., Seifrabie M.A. (2018). Further Evidence of the Positive Influence of Repetitive Transcranial Magnetic Stimulation on Speech and Language in Patients with Aphasia after Stroke: Results from a Double-Blind Intervention with Sham Condition. Neuropsychobiology.

[152] Ren C., Zhang G., Xu X., Hao J., Fang H., Chen P., Li Z., Ji Y., Cai Q., Gao F. (2019). The Effect of rTMS over the Different Targets on Language Recovery in Stroke Patients with Global Aphasia: A Randomized Sham-Controlled Study. BioMed Res. Int..

[153] Zhang H., Chen Y., Hu R., Yang L., Wang M., Zhang J., Lu H., Wu Y., Du X. (2017). rTMS treatments combined with speech training for a conduction aphasia patient: A case report with MRI study. Medicine.

[154] Hara T., Abo M., Kobayashi K., Watanabe M., Kakuda W., Senoo A. (2015). Effects of Low-Frequency Repetitive Transcranial Magnetic Stimulation Combined with Intensive Speech Therapy on Cerebral Blood Flow in Post-Stroke Aphasia. Transl. Stroke Res..

[155] Darkow R., Martin A., Würtz A., Flöel A., Meinzer M. (2017). Transcranial direct current stimulation effects on neural processing in post-stroke aphasia. Hum. Brain Mapp..

[156] Fridriksson J., Richardson J.D., Baker J.M., Rorden C. (2011). Transcranial Direct Current Stimulation Improves Naming Reaction Time in Fluent Aphasia. Stroke.

[157] Fridriksson J., Rorden C., Elm J., Sen S., George M.S., Bonilha L. (2018). Transcranial Direct Current Stimulation vs Sham Stimulation to Treat Aphasia After Stroke. JAMA Neurol..

[158] Pestalozzi M.I., Di Pietro M., Gaytanidis C.M., Spierer L., Schnider A., Chouiter L., Colombo F., Annoni J.-M., Jost L.B. (2018). Effects of Prefrontal Transcranial Direct Current Stimulation on Lexical Access in Chronic Poststroke Aphasia. Neurorehabilit. Neural Repair.

[159] Da Silva F.R., Mac-Kay A.P.M.G., Chao J.C., Dos Santos M.D., Gagliadi R.J. (2018). Estimulação transcraniana por corrente contínua: Estudo sobre respostas em tarefas de nomeação em afásicos. Codas.

[160] Feil S., Eisenhut P., Strakeljahn F., Müller S., Nauer C., Bansi J., Weber S., Liebs A., Lefaucheur J.-P., Kesselring J. (2019). Left Shifting of Language Related Activity Induced by Bihemispheric tDCS in Postacute Aphasia Following Stroke. Front. Neurosci..

[161] Marangolo P., Fiori V., Sabatini U., De Pasquale G., Razzano C., Caltagirone C., Gili T. (2016). Bilateral Transcranial Direct Current Stimulation Language Treatment Enhances Functional Connectivity in the Left Hemisphere: Preliminary Data from Aphasia. J. Cogn. Neurosci..

[162] Marangolo P., Fiori V., Caltagirone C., Pisano F., Priori A. (2018). Transcranial Cerebellar Direct Current Stimulation Enhances Verb Generation but Not Verb Naming in Poststroke Aphasia. J. Cogn. Neurosci..

[163] Spielmann K., van de Sandt-Koenderman W.M.E., Heijenbrok-Kal M.H., Ribbers G.M. (2018). Transcranial Direct Current Stimulation Does Not Improve Language Outcome in Subacute Poststroke Aphasia. Stroke.

[164] Takizawa C., Gemmell E., Kenworthy J., Speyer R. (2016). A Systematic Review of the Prevalence of Oropharyngeal Dysphagia in Stroke, Parkinson’s Disease, Alzheimer’s Disease, Head Injury, and Pneumonia. Dysphagia.

[165] Jean A. (2001). Brain Stem Control of Swallowing: Neuronal Network and Cellular Mechanisms. Physiol. Rev..

[166] Wilmskoetter J., Daniels S.K., Miller A.J. (2020). Cortical and Subcortical Control of Swallowing—Can We Use Information From Lesion Locations to Improve Diagnosis and Treatment for Patients With Stroke?. Am. J. Speech-Lang. Pathol..

[167] Daniels S.K., Pathak S., Mukhi S.V., Stach C.B., Morgan R.O., Anderson J.A. (2017). The Relationship Between Lesion Localization and Dysphagia in Acute Stroke. Dysphagia.

[168] Kubis N. (2016). Non-Invasive Brain Stimulation to Enhance Post-Stroke Recovery. Front. Neural Circuits.

[169] Zhang C., Zheng X., Lu R., Yun W., Yun H., Zhou X. (2019). Repetitive transcranial magnetic stimulation in combination with neuromuscular electrical stimulation for treatment of post-stroke dysphagia. J. Int. Med Res..

[170] Liao X., Xing G., Guoqiang X., Jin Y., Tang Q., He B., A McClure M., Liu H., Chen H., Mu Q. (2017). Repetitive transcranial magnetic stimulation as an alternative therapy for dysphagia after stroke: A systematic review and meta-analysis. Clin. Rehabil..

[171] Park E., Kim M.S., Chang W.H., Oh S.M., Kim Y.K., Lee A. (2017). Effects of Bilateral Repetitive Transcranial Magnetic Stimulation on Post-Stroke Dysphagia. Brain Stimul..

[172] Pisegna J.M., Kaneoka A., Pearson W.G., Kumar S., Langmore S.E. (2016). Effects of non-invasive brain stimulation on post-stroke dysphagia: A systematic review and meta-analysis of randomized controlled trials. Clin. Neurophysiol..

[173] Li Y., Feng H., Li J., Wang H., Chen N., Yang J. (2020). The effect of transcranial direct current stimulation of pharyngeal motor cortex on swallowing function in patients with chronic dysphagia after stroke. Medicine.

[174] Cheng I., Hamdy S. (2021). Current perspectives on the benefits, risks, and limitations of noninvasive brain stimulation (NIBS) for post-stroke dysphagia. Expert Rev. Neurother..

[175] Wang Z., Song W.-Q., Wang L. (2017). Application of noninvasive brain stimulation for post-stroke dysphagia rehabilitation. Kaohsiung J. Med Sci..

[176] Rofes L., Vilardell N., Clavé P. (2013). Post-stroke dysphagia: Progress at last. Neurogastroenterol. Motil..

[177] Pendlebury S.T., Mariz J., Bull L., Mehta Z., Rothwell P.M. (2013). Impact of Different Operational Definitions on Mild Cognitive Impairment Rate and MMSE and MoCA Performance in Transient Ischaemic Attack and Stroke. Cerebrovasc. Dis..

[178] Liu M., Bao G., Bai L., Yu E. (2021). The role of repetitive transcranial magnetic stimulation in the treatment of cognitive impairment in stroke patients: A systematic review and meta-analysis. Sci. Prog..

[179] Miniussi C., Cappa S., Cohen L.G., Floel A., Fregni F., Nitsche M.A., Oliveri M., Pascual-Leone A., Paulus W., Priori A. (2008). Efficacy of repetitive transcranial magnetic stimulation/transcranial direct current stimulation in cognitive neurorehabilitation. Brain Stimul..

[180] Begemann M.J., Brand B.A., Ćurčić-Blake B., Aleman A., Sommer I.E. (2020). Efficacy of non-invasive brain stimulation on cognitive functioning in brain disorders: A meta-analysis. Psychol. Med..

[181] Liu Y., Chen Z., Luo J., Yin M., Li L., Yang Y., Zheng H., Liang Z., Hu X. (2021). Explore combined use of transcranial direct current stimulation and cognitive training on executive function after stroke. J. Rehabil. Med..

[182] Van Lieshout E.C.C., Van Hooijdonk R.F., Dijkhuizen R.M., Visser-Meily J.M.A., Nijboer T.C.W. (2019). The Effect of Noninvasive Brain Stimulation on Poststroke Cognitive Function: A Systematic Review. Neurorehabilit. Neural Repair.

[183] Elsner B., Kugler J., Pohl M., Mehrholz J. (2020). Transcranial direct current stimulation (tDCS) for improving activities of daily living, and physical and cognitive functioning, in people after stroke. Cochrane Database Syst. Rev..

[184] Yan R.-B., Zhang X.-L., Li Y.-H., Hou J.-M., Chen H., Liu H.-L. (2020). Effect of transcranial direct-current stimulation on cognitive function in stroke patients: A systematic review and meta-analysis. PLoS ONE.

[185] Chang W.H., Bang O.Y., Shin Y.-I., Lee A., Pascual-Leone A., Kim Y.-H. (2014). BDNF Polymorphism and Differential rTMS Effects on Motor Recovery of Stroke Patients. Brain Stimul..

[186] Houben M., Chettouf S., Van Der Werf Y.D., Stins J. (2021). Theta-burst transcranial magnetic stimulation for the treatment of unilateral neglect in stroke patients: A systematic review and best evidence synthesis. Restor. Neurol. Neurosci..

[187] Cazzoli D., Müri R.M., Hess C.W., Nyffeler T. (2010). Treatment of hemispatial neglect by means of rTMS—A review. Restor. Neurol. Neurosci..

[188] Yang N.Y., Fong K.N., Li-Tsang C.W., Zhou D. (2017). Effects of repetitive transcranial magnetic stimulation combined with sensory cueing on unilateral neglect in subacute patients with right hemispheric stroke: A randomized controlled study. Clin. Rehabil..

[189] Zhang R.-G., Liu S.-X., Wang F.-Y., Ma X.-C., Yang Y.-H. (2017). Treatment of Unilateral Neglect using Repetitive Transcranial Magnetic Stimulation (rTMS) and Sensory Cueing (SC) in Stroke Patients. Sichuan da xue xue bao. Yi xue ban J. Sichuan Univ. Med Sci. Ed..

[190] Làdavas E., Giulietti S., Avenanti A., Bertini C., Lorenzini E., Quinquinio C., Serino A. (2015). a-tDCS on the ipsilesional parietal cortex boosts the effects of prism adaptation treatment in neglect. Restor. Neurol. Neurosci..

[191] Sunwoo H., Kim Y.-H., Chang W.H., Noh S., Kim E.-J., Ko M.-H. (2013). Effects of dual transcranial direct current stimulation on post-stroke unilateral visuospatial neglect. Neurosci. Lett..

[192] Luvizutto G.J., Rizzati G.R.S., Fogaroli M.O., Rodrigues R.T., Ribeiro P.W., Nunes H.R.D.C., Braga G.P., da Costa R.D.M., Bazan S.G.Z., Resende L.A.D.L. (2016). Treatment of unilateral spatial neglect after stroke using transcranial direct current stimulation (ELETRON trial): Study protocol for a randomized controlled trial. Trials.

[193] Fan J., Li Y., Yang Y., Qu Y., Li S. (2018). Efficacy of Noninvasive Brain Stimulation on Unilateral Neglect After Stroke. Am. J. Phys. Med. Rehabil..

[194] Kashiwagi F.T., El Dib R., Gomaa H., Gawish N., Suzumura E.A., da Silva T.R., Winckler F., DE Souza J.T., Conforto A.B., Luvizutto G.J. (2018). Noninvasive Brain Stimulations for Unilateral Spatial Neglect after Stroke: A Systematic Review and Meta-Analysis of Randomized and Nonrandomized Controlled Trials. Neural Plast..

[195] Longley V., Hazelton C., Heal C., Pollock A., Woodward-Nutt K., Mitchell C., Pobric G., Vail A., Bowen A. (2021). Non-pharmacological interventions for spatial neglect or inattention following stroke and other non-progressive brain injury. Cochrane Database Syst. Rev..

[196] Taylor-Rowan M., Momoh O., Ayerbe L., Evans J.J., Stott D.J., Quinn T.J. (2019). Prevalence of pre-stroke depression and its association with post-stroke depression: A systematic review and meta-analysis. Psychol. Med..

[197] Mitchell A.J., Sheth B., Gill J., Yadegarfar M., Stubbs B., Yadegarfar M., Meader N. (2017). Prevalence and predictors of post-stroke mood disorders: A meta-analysis and meta-regression of depression, anxiety and adjustment disorder. Gen. Hosp. Psychiatry.

[198] Villa R.F., Ferrari F., Moretti A. (2018). Post-stroke depression: Mechanisms and pharmacological treatment. Pharmacol. Ther..

[199] Loubinoux I., Kronenberg G., Endres M., Schumann-Bard P., Freret T., Filipkowski R.K., Kaczmarek L., Popa-Wagner A. (2012). Post-stroke depression: Mechanisms, translation and therapy. J. Cell. Mol. Med..

[200] Medeiros G.C., Roy D., Kontos N., Beach S.R. (2020). Post-stroke depression: A 2020 updated review. Gen. Hosp. Psychiatry.

[201] Shen X., Liu M., Cheng Y., Jia C., Pan X., Gou Q., Liu X., Cao H., Zhang L. (2017). Repetitive transcranial magnetic stimulation for the treatment of post-stroke depression: A systematic review and meta-analysis of randomized controlled clinical trials. J. Affect. Disord..

[202] Liu C., Wang M., Liang X., Xue J., Zhang G. (2019). Efficacy and Safety of High-Frequency Repetitive Transcranial Magnetic Stimulation for Poststroke Depression: A Systematic Review and Meta-analysis. Arch. Phys. Med. Rehabil..

[203] Shao D., Zhao Z., Zhang Y., Zhou X., Zhao L., Dong M., Xu F., Xiang Y., Luo H. (2021). Efficacy of repetitive transcranial magnetic stimulation for post-stroke depression: A systematic review and meta-analysis of randomized clinical trials. Braz. J. Med Biol. Res..

[204] Palm U., Ayache S., Padberg F., Lefaucheur J.-P. (2016). La stimulation transcrânienne à courant continu (tDCS) dans la dépression: Bilan de près d’une décennie de recherche clinique. L’Encéphale.

[205] Valiengo L.C.L., Goulart A.C., de Oliveira J.F., Benseñor I.M., A Lotufo P., Brunoni A.R. (2017). Transcranial direct current stimulation for the treatment of post-stroke depression: Results from a randomised, sham-controlled, double-blinded trial. J. Neurol. Neurosurg. Psychiatry.

[206] Gupta A., Adnan M. (2018). Hypomania Risk in Noninvasive Brain Stimulation. Cureus J. Med. Sci..

[207] Baig S.S., Kamarova M., Ali A., Su L., Dawson J., Redgrave J.N., Majid A. (2022). Transcutaneous vagus nerve stimulation (tVNS) in stroke: The evidence, challenges and future directions. Auton. Neurosci..

[208] Elsner B., Kwakkel G., Kugler J., Mehrholz J. (2017). Transcranial direct current stimulation (tDCS) for improving capacity in activities and arm function after stroke: A network meta-analysis of randomised controlled trials. J. Neuroeng. Rehabil..

[209] Van Lieshout E.C.C., Van Der Worp H.B., Visser-Meily J.M.A., Dijkhuizen R.M. (2019). Timing of Repetitive Transcranial Magnetic Stimulation Onset for Upper Limb Function After Stroke: A Systematic Review and Meta-Analysis. Front. Neurol..

[210] Yoon B.-W., Ryu S., Lee S.-H., Kim S.U. (2016). Human neural stem cells promote proliferation of endogenous neural stem cells and enhance angiogenesis in ischemic rat brain. Neural Regen. Res..

[211] Palmer J.A., Wolf S.L., Borich M.R. (2018). Paired associative stimulation modulates corticomotor excitability in chronic stroke: A preliminary investigation. Restor. Neurol. Neurosci..

[212] Kuo I.-J., Tang C.-W., Tsai Y.-A., Tang S.-C., Lin C.-J., Hsu S.-P., Liang W.-K., Juan C.-H., Zich C., Stagg C.J. (2020). Neurophysiological signatures of hand motor response to dual-transcranial direct current stimulation in subacute stroke: A TMS and MEG study. J. Neuroeng. Rehabil..

[213] Conner J.M., Bohannon A., Igarashi M., Taniguchi J., Baltar N., Azim E. (2021). Modulation of tactile feedback for the execution of dexterous movement. Science.

[214] Downar J., Daskalakis Z.J. (2013). New Targets for rTMS in Depression: A Review of Convergent Evidence. Brain Stimul..

[215] Braga M., Barbiani D., Andani M.E., Villa-Sánchez B., Tinazzi M., Fiorio M. (2021). The Role of Expectation and Beliefs on the Effects of Non-Invasive Brain Stimulation. Brain Sci..

[216] Raco V., Bauer R., Tharsan S., Gharabaghi A. (2016). Combining TMS and tACS for Closed-Loop Phase-Dependent Modulation of Corticospinal Excitability: A Feasibility Study. Front. Cell. Neurosci..

[217] Andani M.E., Villa-Sánchez B., Raneri F., Dametto S., Tinazzi M., Fiorio M. (2020). Cathodal Cerebellar tDCS Combined with Visual Feedback Improves Balance Control. Cerebellum.

